# Predictive Role of Depressive Symptoms on Frailty and its Components in Chinese Middle-Aged and Older Adults: a Longitudinal Analysis

**DOI:** 10.21203/rs.3.rs-3821620/v1

**Published:** 2024-01-08

**Authors:** Yuanhao Sun, Xiangdong Li, Haiyang Liu, Yuqing Li, Jiaofeng Gui, Xiaoyun Zhang, Xiaoping Li, Lu Sun, Congzhi Wang, Jing Li, Mingming Liu, Dongmei Zhang, Jingyi Gao, Xuefeng Kang, Yunxiao Lei, Lin Zhang, Ting Yuan

**Affiliations:** Wannan Medical College; the First Affiliated Hospital of Wannan Medical College; Wannan Medical College Y; Wannan Medical College; Wannan Medical College; Wannan Medical College; Wannan Medical College; Wannan Medical College; Wannan Medical College; Wannan Medical College; Wannan Medical College; Wannan Medical College; Wannan Medical College; Wannan Medical College; Wannan Medical College; Wannan Medical College; Wannan Medical College

**Keywords:** cohort study, depressive symptoms, frailty, middle-aged and elderly

## Abstract

**Background:**

To investigate the cross-sectional and longitudinal associations between depressive symptoms and the prevalence of frailty and its components in a nationally representative sample of middle-aged and older Chinese adults.

**Method:**

The China Health and Retirement Longitudinal Study (CHARLS) provided data on 17,104 adults aged ≥ 45 years. Every two years, face-to-face, computer-aided personal interviews (CAPI), and structured questionnaires were used to follow up with the respondents. The Chinese version of the Center for Epidemiologic Studies-Depression Scale (CES-D) was used to evaluate depressive symptoms, and the Fried criteria were used to measure frailty. The odds ratio (OR) and 95% confidence interval (CI) for the cross-sectional connections among depressive symptoms and frailty and its components in the individuals at baseline were analyzed using logistic regression. A Cox proportional hazards analysis was performed using the hazard ratio (HR) and 95% confidence interval for the prospective connection between baseline depressive symptoms and frailty and its component in the participants without frailty at baseline.

**Results:**

At baseline, 11.62% of participants had frailty, and 57.92% had depressive symptoms. In the cross-sectional analysis, depressive symptoms (OR = 5.222, 95%CI 3.665–7.442) were associated with frailty. In the longitudinal analysis, after adjusting for the full set of covariates among participants free of baseline frailty, depressive symptoms were significantly associated with incident frailty during the short term [HR = 2.193 (1.324–3.631)] and the long term [HR = 1.926 (1.021–3.632)]. Meanwhile, depressive symptoms were associated with an increased risk of weakness [HR = 1.990 (1.250–3.166)], slowness [HR = 1.395 (1.044–1.865)], and exhaustion [HR = 2.827 (2.150–3.719)] onset during the short-term. Depressive symptoms were associated with an increased risk of exhaustion [HR = 2.869 (2.004–4.109)] onset during the long-term.

**Conclusion:**

Among middle-aged and older adults, depressive symptoms could predict frailty during 2 years of follow-up and 4 years of follow-up. When considering potential confounding factors, depressive symptoms were considered a predictor of weakness, slowness, and exhaustion. Interventions aimed at preventing depressive symptoms may be beneficial in reducing frailty and its components.

## BACKGROUND

Frailty is defined by a loss of biological reserves, a failure of homeostasis mechanisms, and vulnerability to physical decompensation after minor influences or stresses [1]. A common biological syndrome model of frailty consists of exhaustion, weakness, low physical activity, slowness, and weight loss. Pre-frailty occurs when one or two of these factors are present, while frailty occurs when three or more are present [2]. As people age, they become more fragile, which increases their risk of several negative health consequences, including hospitalization, falls, and even death [3]. Depression is a common chronic medical illness that can impact one’s mood, thoughts, and physical health [4]. It is characterized by a lack of energy, a low mood, insomnia, sadness, and an inability to enjoy life [4]. In most clinical contexts, depression is assessed as a single condition or severity continuum. This combines varied symptomatology and reduces clinically meaningful information that is critical to diagnosis and treatment [5]. Depression in the elderly contributes to both dementia and a decline in functional ability [6]. Serious consequences like happiness, disability, and an increase in the load on families and society are linked to depressive symptoms [7]. Lifetime prevalence estimates (populational mean 13%) differ significantly between nations [8]. Depression has a prevalence of 10–20% in older adults [9], while it is more common when it comes to women [8], people with lower socioeconomic status [10], and people with less education [11]. Fiske et al. reported that depression is one of the most common mental diseases resulting in disability in late life [6]. According to a meta-analysis, the prevalence of depressive symptomatology is 17.1% in people 75 years of age and older and 19.5% in those 50 years of age and older [12]. The prevalence of geriatric depression disorders ranges from 12.7–33.8% in Asian nations [13]. Major depression is present in older adults at a rate of 4.6–9.3%, while subthreshold depression is two to three times more common than major depression [9]. In Taiwan, the major and minor depression prevalence rates for persons 55 years of age or older are approximately 1.5% and 3.7%, respectively [14].

Mounting evidence demonstrates that depression could be associated with frailty. In a cross-sectional study by Jung et al. involving 382 participants, depressive people were more likely to be frail when compared to those who did not have depression (OR = 5.25, 95% CI, 2.55–10.83) [15]. Older adults with depression have a higher prevalence of physical fragility than those without depression [16]. According to a 1.5-year cohort study involving 1602 Germans, the prevalence of frailty rose along with the growing prevalence of depression [17]. A recent meta-analysis, which combines four cohorts and ten cross-sectional observational studies, involving 84,351 community elderly, confirmed that older adults can become frail by depression and that older men with depression are more likely to become frail than their female counterparts [18]. In the Geriatric Clinic of “Dr. C. I. Parhon” Hospital, a retrospective study involving 411 patients found that frailty is positively correlated with depression [19]. Prospective studies of the connection between depression and incident frailty also showed that depression may raise the risk of frailty [20]. In longitudinal research, frailty and pre-frailty were linked to a 2.31-fold and 1.58-fold higher risk of incidence of depressive symptoms, respectively, compared with no frail individuals, after controlling for sociodemographic variables (e.g., age, gender, alcohol intake, smoking, etc.) [21]. Soysal et al.’s observations indicate that frailty and depression are both risk factors for the occurrence of each other [22]. Fugate Woods et al. indicated that the risk factors of frailty may result in functional dependence, or disability, and thus lead to depression [23]. Another review, including cross-sectional (n = 16) and longitudinal studies (n = 23) deemed that frailty and its components are risk factors for depression symptoms [24]. A cross-sectional examination of one prospective cohort of researchers revealed a correlation between depression and social frailty [25]. Additionally, according to two longitudinal investigations including 4852 older persons, frailty at baseline raised the risk of incident depression by about 90 percent [26, 27].

The meta-analysis emphasized the possible negative impact of depression on frailty and included both cohort and cross-sectional studies. The influence of depression on frailty and its components over a period of years in various research studies was not taken into account. Furthermore, in the meta-analysis, most participants were Western. Therefore, more research on middle-aged and older people in Asian countries is required to find the connection between depressive symptoms and the influence of frailty in Asian participants. In order to close these gaps, this research utilized 4-year longitudinal data from a nationally representative sample of Chinese participants who were 45 years of age or older. The research investigated the association between depressive symptoms and the incidence of frailty and its components during the 2 and 4-year internal studies. Additionally, by adjusting for relevant confounders, this research investigates the stability of the relationship between depressive symptoms, frailty, and its components.

## Materials and methods

### Study participants

**Figure 1:**
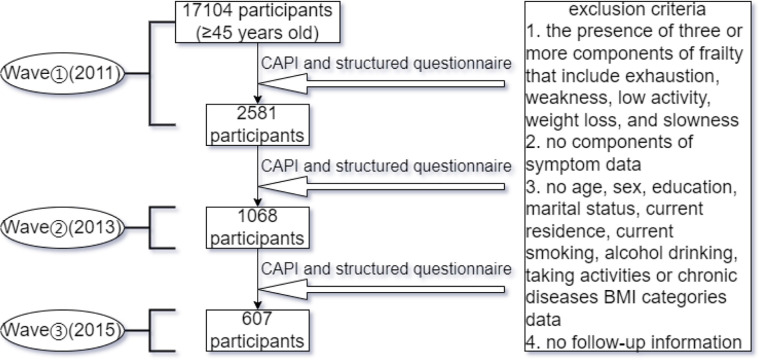
Study procedure CAPI: computer-aided personal interviews

The China Health and Retirement Longitudinal Study (CHARLS), which is a nationally representative longitudinal study of Chinese citizens aged 45 and above and their spouses, provided us with the data. In 2011, 17104 people aged 45 and older were included in the CHARLS cohort (Wave 1). Data collection then took place in 2013 (Waves 2) and 2015 (Waves 3). Every two years, face-to-face, computer-aided personal interviews (CAPI), and structured questionnaires will be used to follow up with the respondents. Data from individuals who took part in Waves 1, 2, and 3 were used in this study. At baseline, the exclusion criteria of the research were: (1) the presence of three or more components of frailty that include exhaustion, weakness, low activity, weight loss, and slowness; (2) no depressive symptoms data; (3) no age, sex, education, marital status, current residence, current smoking, alcohol drinking, taking activities, chronic diseases, or BMI categories data; and (4) no follow-up information. There were 2581 people who finished both baselines, and the number of follow-up surveys was 1068 for the short term (two years, 2011–2013) and 607 for the long term (four years, 2011–2015)(Fig. 1).

### Depressive symptom

The Center for Epidemiologic Studies-Depression Scale (CES-D) was applied in the research to measure depression symptoms in the last week [28, 29]. The scale has good content validity and structure validity, which is the Chinese version. The scale contains 10 items, which are divided into 3 dimensions, including depressed affect for 3 items, somatic symptoms for 5 items, and positive affect for 2 items. Each item used a four-point Likert scale coded from 0 (seldom or none of the time) to 3 (all of the time). 5 and 8 items are negatively stated; other items are positively stated. The total points score on the scale ranges from 0 to 30, and a higher score indicates greater degrees of depressive symptoms. As recommended by Andresen, the depressed symptom-total score ≥10 was established using harmonized criterion cutoff values. The Cronbach’s alpha value for this section was 0.861.

CAPI: computer-aided personal interviews

### Frailty assessment

Nowadays, the Fried model is the most commonly used and accepted as the standard for frailty evaluation [2]. The information from the CHARLS was used to modify it. In this conceptual model, frailty is reflected across five components: weakness, slowness, weight loss, exhaustion, and low activity. In this research, the following definitions and assessments were made of the five frailty components. Frailty refers to meeting three or more out of five components.

1. Weakness: According to Fried et al., the body mass index (BMI) and sex cutoff were used to define weakness as maximum grip strength [2]. Using a dynamometer, the maximal handgrip strength was measured three times on each side; the best measurement was selected in our analysis. For medical reasons, people who were unable to do the handgrip strength test were deemed weakness.
2. Slowness: Slowness means being below the 20th sex-specific percentile. A Timed Up and Go (TUG) test was used to measure gait speed [30]. The TUG test required research participants to get out of an armchair, walk three meters, and then get back in and sit down. The test started when the individual’s back left the armchair, and it ended when their buttocks made contact with the chair’s seat once more.
3. Weight loss: weight loss refers to the weight that has decreased by 5 kg or current body mass index (BMI) ≤ 18.5 kg/m^2^ during the last 12 months [31, 32].
4. Exhaustion: Exhaustion refers to the response to two items from the CES-D: (1) “I thought that everything I did was an effort”; and (2) “I could not get going.” If the participants feel tired all of the time, at least 3 or 4 days per week, they would be determined to be positive [2].
5. Low activity: The physical activity questions in the Health Survey for England were taken from a validated physical activity interview [33]. A question regarding the frequency of moderate activity (such as dancing, walking at a moderate pace, cleaning the car, gardening, floor, or stretching exercises) was answered by the participants. Low physical activity was defined as answering “hardly ever” or “never”. It was different from that proposed by Fried et al. [2]. In Xu’s research, low physical activity has been evaluated using similar treatment variables [34].

### Body measurement

In 1835, Adolphe Quetelet, a Belgian mathematician, astronomer, and statistician, established the concept of body mass index (BMI), which is determined by dividing body weight in kilograms by the square of height in meters (kg/m^2^) [35]. These days, BMI is often used in part because it is easily measured and consistently recorded in patient medical records for the routine characterization of weight status in epidemiology, clinical nutrition, and research [36]. Participants are categorized as underweight (BMI < 18.5kg/m^2^), normal (18.5–24kg/m^2^), overweight (24–28kg/m^2^), and obese (≥ 28kg/m^2^) [37, 38].

### Covariates

Age, sex (male and female), educational levels, marital status, current residence (rural and urban), current smoking, alcohol drinking, taking activities, chronic diseases, BMI at baseline, and entry wave (Waves 1, 2, and 3) were incorporated as covariates in the present research. (1) There were four age groups: under 45–54, 55–64, 65–74, and over 75 years old. (2) Educational levels, including illiterate (no formal education), less than elementary school (did not complete primary school but were able to write and graduate from home school, elementary school, or middle school), high school, and above vocational school (graduate from a two- or three-year college, graduate from an undergraduate college, graduate from a post-graduate). (3) There were two categories for marital status: single (not married, separated, divorced, or widowed) and married. (4) There were two categories for current residence: rural and urban. (5) There were three categories for current smoking: current smoker, former smoker, and never smoker. (6) There were three categories for alcohol drinking: never drinkers, less than once a month, and more than once a month. (7) There were two categories for taking activities (interacted with friends/helped family, friends or neighbors who don’t live with you and didn’t charge you for it/visited a club for sports, social, or another form/went to a community club, played chess, played cards, or played mahjong/participated in a community-related organization/done charity or voluntary work/looked after an ill or disabled adult who was not staying with you and didn’t pay you for your assistance/participated in a training or educational activity/stock investment /used the Internet): as ever (at least once a month) and never. (8) Chronic diseases were defined according to whether a doctor told individuals they had any of the following conditions: hypertension, diabetes or high blood sugar, cancer or malignant tumor (excluding minor skin cancers), dyslipidemia, chronic lung diseases, liver disease (except fatty liver, tumors, and cancer), kidney disease (except for tumor or cancer), heart attack, angina, coronary heart disease, congestive heart failure, or other heart problems, stomach or other digestive disease (except for tumor or cancer), stroke, memory-related disease, emotional, nervous, or psychiatric problems, arthritis or rheumatism, and asthma. Among the 14 common chronic diseases, which have a range of 0 to 14, a continuous variable is used to represent the presence of chronic health issues [39]. There were three categories for chronic disease, with the numbers of the condition being 0, 1–2, and 3–14, respectively.

### Statistical analysis

The study used IBM SPSS version 25.0 (Chicago, IL, USA) for all statistical analyses. The distribution of categorical variables was expressed as frequencies and percentages and analyzed by chi-square. The study used logistic regression to analyze the odds ratio (OR) and 95% confidence interval (CI) for the cross-sectional associations of depressive symptoms with frailty in the participants at baseline. Frailty was analyzed as a binary dependent variable (no-frail and frail), and covariates were included in the regression models in steps. Model 1 included depressive symptoms only; Model 2 additionally included social-demographic characteristics (age, sex, educational levels, marital status, and living place); Model 3 additionally included health behaviors and conditions (current smoking, alcohol drinking, activities, and chronic diseases); and Model 4 further included body measure (BMI). The study used the hazard ratio (HR) to conduct a Cox proportional hazards analysis and a 95% confidence interval (CI) for the prospective associations of baseline depression symptoms in the participants without frailty at baseline. Cross-sectional analysis approaches were used to model covariates. P < 0.05 was considered statistically significant for all statistical analyses.

## Results

Table 1 shows the baseline characteristics of participants according to the level of frailty. The mean age of participants was 61.06 ± 10.12; 62.03% were female; 84.00% were married; and 91.75% were living in rural areas. 8.99% were former smokers, and 24.60% were current smokers; 7.09% were drinking less than once a month, and 19.33% were drinking more than once a month; 49.24% were taking activities; 51.34% had 1–2 chronic diseases, and 27.16% had 3–14 chronic diseases. The frequency of frailty was 11.62%. The differences among participants with or without frailty were observed in the distribution of age, sex, educational levels, marital status, live place, current smoking, alcohol drinking, activities, chronic diseases, and BMI categories.

Table 2 shows the baseline characteristics of participants according to the level of depression. A total of 2581 robust participants (42.08%) and depressive symptoms(57.92%) at baseline were included in the cross-sectional analysis. The differences in depressive symptoms were observed in the distribution of age, sex, educational levels, marital status, live place, current smoking, alcohol drinking, activities, chronic diseases, and BMI categories.

Table 3 shows baseline characteristics classified according to subsequent onset of frailty. In the short-term (2 years from 2011 to 2013), participants who developed frailty were more likely to be female and to live in rural areas. They tend to never smoke. In the long-term (4 years from 2011 to 2015), participants who developed frailty were also more likely to be female and to take no part in activities.

Table 4 shows the cross-sectional relationship between depressive symptoms and frailty at baseline. Depressive symptoms (OR = 5.222, 95%CI 3.665–7.422) were significantly associated with frailty after adjusting for age, sex, educational levels, marital status, live place, current smoking, alcohol drinking, activities, chronic diseases, and BMI (adjusted model 4). In the depressive symptoms, after adjusting for the full set of covariates, depression was associated with weakness (OR = 2.037, 95%CI 1.510–2.748), slowness (OR = 1.858, 95%CI 1.528–2.259), weight loss (OR = 1.531, 95%CI 1.170–2.004), and exhaustion (OR = 12.140, 95%CI 9.903–14.882). However, depressive symptoms were not associated with low physical activity (OR = 1.207, 95%CI 0.957–1.521).

Table 5 shows the prospective associations between baseline depressive symptoms and frailty at 2- and 4-year follow-up survey in the participants without frailty at baseline. Firstly, in crude analysis, depressive symptoms were significantly associated with incident frailty during the short-term (OR = 2.148, 95%CI 1.323–3.488). Secondly, in crude analysis, the depressive symptoms (OR = 2.032, 95%CI 1.107–3.731) were significantly associated with incident frailty during the long-term. Thirdly, after adjusting for age, sex, educational levels, marital status, live place, current smoking, alcohol drinking, activities, chronic diseases, and BMI, the depressive symptoms (OR = 2.193, 95%CI 1.324–3.631) were significantly associated with incident frailty during the short-term. Lastly, after adjusting for the full set of covariates, the HR for depressive symptoms was 1.926 (95%CI 1.021–3.632) during the long-term.

Table 6 shows the association between depressive symptoms and components of frailty in 2011–2013, not frailty at baseline. Firstly, in crude analysis, frailty (OR = 2.148, 95%CI 1.323–3.488) risk was increased for the depressive symptoms during the short-term. Secondly, depressive symptoms were significantly associated with incident frailty which included weakness, slowness, weight loss, exhaustion and low activity [weakness: HR = 2.003 (1.279, 3.316), slowness: HR = 1.375 (1.045, 1.809), weight loss: HR = 1.517 (1.031, 2.232), exhaustion: HR = 2.878 (2.212, 3.744), low activity: HR = 0.648 (0.455, 0.924)] during the short-term. Thirdly, after adjusting for age, sex, educational levels, marital status, live place, current smoking, alcohol drinking, activities, chronic diseases, and BMI, depressive symptoms were significantly associated with frailty (OR = 2.193, 95%CI 1.324–3.631), weakness (OR = 1.990, 95%CI 1.250–3.166), slowness (OR = 1.395, 95%CI 1.044–1.865), and exhaustion (OR = 2.827, 95%CI 2.150–3.719) during the short-term. However, depressive symptoms were not significantly associated with weight loss and low activity [weight loss: HR = 1.374 (0.923, 2.046), low activity: HR = 0.695, (0.481, 1.004)].

Table 7 shows the association between depressive symptoms and components of frailty in 2011–2015, not frailty at baseline. Firstly, in crude analysis, frailty (OR = 2.032, 95% CI 1.107–3.731) risk was increased for the depressive symptoms during the long-term. Secondly, depressive symptoms were significantly associated with frailty (OR = 2.032, 95%CI 1.107–3.731) and exhaustion (OR = 2.904, 95%CI 2.052–4.111) during the long-term. However, depressive symptoms were not significantly associated with weakness, slowness, weight loss, or low activity [weakness: HR = 1.107 (0.727, 1.685), slowness: HR = 0.916 (0.637, 1.316), weight loss: HR = 1.620 (0.866, 3.029), low activity: HR = 0.823 (0.519, 1.306)]. Thirdly, after adjusting for age, sex, educational levels, marital status, live place, current smoking, alcohol drinking, activities, chronic diseases, and BMI, depressive symptoms was significantly associated with frailty (OR = 1.926, 95%CI 1.021–3.632) and exhaustion (OR = 2.869, 95%CI 2.004–4.109) during the long-term. However, depressive symptoms were not significantly associated with weakness, slowness, weight loss, or low activity [weakness: HR = 0.983 (0.630, 1.534), slowness: HR = 0.845 (0.576, 1.239), weight loss: HR = 1.503 (0.666, 3.393), low activity: HR = 0.839 (0.520, 1.353)].

## DISCUSSION

Previous researches have reported differences in the relationship between depressive symptoms and the incidence of frailty [15–27]. Meanwhile, there have been few findings regarding the relationship between the middle-aged and the elderly in China. The cross-sectional and longitudinal associations between depressive symptoms and frailty and its components were described in this research. First, it has been found that depressive symptoms at baseline were related to frailty and its components (weakness, slowness, weight loss, and exhaustion). Secondly, depressive symptoms at baseline were significantly associated with the onset of frailty after two years of follow-up. Among specific criteria, weakness, slowness, and exhaustion were significant independent predictors of future frailty. Lastly, depressive symptoms at baseline were significantly associated with the onset of frailty after four years of follow-up. Among specific criteria, exhaustion was a significant independent predictor of future frailty. Depression symptoms should be evaluated for prevention, as they may be a potential future risk factor for future frailty.

Several studies focused more on the possible negative impact of depression on frailty [15–21]. To date, a limited amount of research has explored the influence of depression on frailty and its components over a period of years. At the same time, in the meta-analysis, most participants were Western. It is essential to find the connection between depressive symptoms and the influence of frailty in Asian participants, who are middle-aged and older. This research aimed to investigate the association between depressive symptoms and the incidence of frailty and its components during the 2 and 4-year internal studies. Several findings have shown that depressive symptoms have been related to an increased risk of frailty and its similar components, such as cognitive decline, reduced social and physical activity (e.g., as a result of muscular atrophy), memory consolidation, mental flexibility and somatic health decline [40–43]. Soysal’s research also found that depressive symptoms often lead to weight loss, slow gait speed, poor social relationships, and malnutrition [22]. In addition, Veronese et al. deemed that depressive symptoms can lead to fatigue, weakness, and mobility impairments [44]. These factors may raise the risk of frailty and increased mortality during a five-year period. The above studies are partly in accordance with this research. With regard to the depressive symptoms, we found depressive symptoms were related to an increased risk of incident frailty, weakness, slowness, and exhaustion after 2 years of follow-up in older adults without baseline frailty. Moreover, depressive symptoms were significantly associated with the onset of frailty and exhaustion after 4 years of follow-up. Results from the present longitudinal data in this research indicate that depressive symptoms are associated with an increased risk of incident frailty after 2 and 4 years of follow-up in the middle-aged and elderly aged 45–96 years. The differences between our research and the previously referenced studies in the literature could be explained by some hypotheses. First, differences in evaluation tools for frailty and depressive symptoms, covariates, and length of follow-up could play an important role. There are 10-item, 15-item, and 20-item Epidemiologic Studies-Depression Scales. This research used a 10-item Epidemiologic Studies-Depression Scale to evaluate depressive symptoms. Second, the transcultural differences, the population, and the data collection may contribute to these differences in the results. Finally, we used the revised Fried’s criteria, which may influence the results [2].

The mechanisms that underline the association between depressive symptoms and the incidence of frailty and its components are still unknown. Some hypotheses could explain the significant association between depressive symptoms and the onset of weakness, slowness, and exhaustion. First, due to the decline in social ties, gait speed, and reduced physical activity, as well as the rise in sedentary lifestyles, weight loss, fall risk, and malnourishment, depression may be predictive of frailty [17, 45]. These factors may also prolong the depressive symptoms, such as sadness, hopelessness, and anhedonia. Second, common risk factors and pathophysiologic pathways may exist. Overlapping mechanisms can partly explain these, such as chronic inflammation, cerebrovascular disease, oxidative stress, hypothalamic-pituitary-adrenal dysfunction, mitochondrial, and axis dysregulation [22, 46–48]. At the same time, subclinical vascular diseases, which result in pre-frontal white-matter hyperintensities in individuals with late-life depressive symptoms, have long been recognized as a critical factor in prefrailty [49]. Third, the level of the inflammatory cytokines, for example, interleukin 6 (IL 6), will be raised in individuals with late-life depressive symptoms [20]. In addition to having a negative impact on central dopaminergic function, inflammatory cytokines are linked to decreased muscular mass and strength; these effects may also cause fatigue and motor slowing [47]. Finally, mitochondrial dysfunction may be a key factor. Patients with depression found reduced ATP (adenosine triphosphate) generation in their muscle biopsies [47]. Peripheral blood mononuclear cells from people with depression showed decreased mitochondrial respiration, which was most closely correlated with the fatigue symptom [47].

For older people with frailty, physical activity is a beneficial intervention. Through possible neurobiological changes, as well as a result of physical and social engagement, it may prevent and manage depression symptoms among older people [47, 50, 51]. Among middle-aged and older adults, interventions aimed at preventing depressive symptoms may be beneficial in reducing frailty and its components.

## STRENGTHES AND LIMITATIONS OF THE STUDY

The research has several strengths. First, the research was based on a nationwide population-based longitudinal study, which included 17104 adults aged 45 and above. It ensures the accuracy of this study and therefore can be considered nationally representative. It means the results may be used for “cause inference”. Second, the measures of depressive symptoms and frailty were widely applied and validated instruments to thoroughly understand the research questions. Finally, it investigated how depression symptoms affected frailty and its components at two distinct intervals. The connection between depressive symptoms and frailty was identified in previous research only at a single interval. It improves our understanding of the short- and long-term effects of depressive symptoms on the incidence of frailty.

Several limitations of this research should be noted. First, during the three waves, when the depressive symptom was evaluated subjectively, it was self-reported. A reporting bias might exist in this. Second, the different methods and cut-off values used for defining depressive symptoms and frailty might lead to the heterogeneity observed. Finally, the duration of the follow-up period is insufficient to gather additional incident outcome cases for a more precise effect estimation. So future studies need to enhance these aspects.

## CONCLUSIONS

Among middle-aged and older adults, depressive symptoms could predict frailty during 2 years of follow-up and 4 years of follow-up. When considering potential confounding factors, depressive symptoms were considered a predictor of weakness, slowness, and exhaustion. Interventions aimed at preventing depressive symptoms may be beneficial in reducing frailty and its components.

## Figures and Tables

**Table 1:** Baseline characteristics of participants according to the level of frail in CHARLS Waves2011 (N, %)

Variables	All Participants (2581)	No-Frail (2281)	Frail (300)	*t/χ^2^*	*P*-value
Age(years)	61.06 ± 10.12	60.43 ±9.86	65.83 ±10.79	−8.820	0.000
Age groups(years)					
45–54	727(28.17)	688(30.16)	39(13.00)	79.175	0.000
55–64	950(36.81)	847(37.13)	103(34.33)		
65–74	620(24.02)	532(23.32)	88(29.33)		
≥75	284(11.00)	214(9.38)	70(23.33)		
Sex					
Male	980(37.97)	897(39.32)	83(27.67)	15.300	0.000
Female	1601(62.03)	1384(60.68)	217(72.33)		
Education					
Illiterate	891(34.52)	740(32.44)	151(50.33)	40.043	0.000
Less than elementary school	1518(58.81)	1378(60.41)	140(46.67)		
High school	102(3.95)	96(4.21)	6(2.00)		
Above vocational school	70(2.71)	67(2.94)	3(1.00)		
Marital status					
Single	413(16.00)	348(15.26)	65(21.67)	8.105	0.004
Married	2168(84.00)	1933(84.74)	235(78.33)		
Current residence					
Rural	2368(91.75)	2085(91.41)	283(94.33)	2.998	0.083
Urban	213(8.25)	196(8.59)	17(5.67)		
Current smoking					
No	1714(66.41)	1498(65.67)	216(72.00)	6.505	0.039
Former smoke	232(8.99)	204(8.94)	28(9.33)		
Current smoke	635(24.60)	579(25.38)	56(18.67)		
Alcohol drinking					
No	1899(73.58)	1654(72.51)	245(81.67)	12.737	0.002
Less than once a month	183(7.09)	164(7.19)	19(6.33)		
More than once a month	499(19.33)	463(20.30)	36(12.00)		
Taking activities					
No	1310(50.76)	1131(49.58)	179(59.67)	10.785	0.001
Yes	1271(49.24)	1150(50.42)	121(40.33)		
Chronic diseases(counts)	1.76 ± 1.49	1.68 ± 1.43	2.38 ± 1.77	−7.679	0.000
Chronic diseases groups(counts)					
0	555(21.50)	517(22.67)	38(12.67)	42.235	0.000
1–2	1325(51.34)	1189(52.13)	136(45.33)		
3–14	701(27.16)	575(25.21)	126(42.00)		
BMI (kg/m^2^)	23.70 ± 4.09	23.76 ± 4.02	23.26 ± 4.55	1.973	0.049
BMI categories					
<18.5	200(7.75)	155(6.80)	45(15.00)	29.021	0.000
18.5–24	1253(48.55)	1112(48.75)	141(47.00)		
24–28	791(30.65)	720(31.57)	71(23.67)		
≥28	337(13.06)	294(12.89)	43(14.33)		
Depressive symptom	11.13 ± 5.08	10.66 ± 4.92	14.72 ± 4.81	−13.782	0.000
No	1086(42.08)	1046(45.86)	40(13.33)	115.07	0.000
Yes	1495(57.92)	1235(54.14)	260(86.67)		

**Table 2:** Baseline characteristics of participants according to the level of Depressive Symptoms in CHARLS Waves2011

Variables	All Participants (2581)	No-depressive symptoms (1086)	Depressive symptoms (1495)	*t/χ^2^*	*P*-value
Age(years)	61.06 ± 10.12	61.67 ± 10.29	60.61 ± 9.98	2.611	0.009
Age groups(years)					
45–54	727(28.17)	298(27.44)	429(28.70)	10.027	0.018
55–64	950(36.81)	371(34.16)	579(38.73)		
65–74	620(24.02)	287(26.43)	333(22.27)		
≥75	284(11.00)	130(11.97)	154(10.30)		
Sex					
Male	980(37.97)	496(45.67)	484(32.37)	47.227	0.000
Female	1601(62.03)	590(54.33)	1011(67.63)		
Education					
Illiterate	891(34.52)	329(30.29)	562(37.59)	44.697	0.000
Less than elementary school	1518(58.81)	647(59.58)	871(58.26)		
High school	102(3.95)	62(5.71)	40(2.68)		
Above vocational school	70(2.71)	48(4.42)	22(1.47)		
Marital status					
Single	413(16.00)	153(14.09)	260(17.39)	5.106	0.024
Married	2168(84.00)	933(85.91)	1235(82.61)		
Current residence					
Rural	2368(91.75)	974(89.69)	1394(93.24)	10.513	0.001
Urban	213(8.25)	112(10.31)	101(6.76)		
Current smoking					
No	1714(66.41)	675(62.15)	1039(69.50)	16.761	0.000
Former smoke	232(8.99)	118(10.87)	114(7.63)		
Current smoke	635(24.60)	293(26.98)	342(22.88)		
Alcohol drinking					
No	1899(73.58)	772(71.09)	1127(75.38)	9.200	0.010
Less than once a month	183(7.09)	74(6.81)	109(7.29)		
More than once a month	499(19.33)	240(22.10)	259(17.32)		
Taking activities					
No	1310(50.76)	529(48.71)	781(52.24)	3.136	0.077
Yes	1271(49.24)	557(51.29)	714(47.76)		
Chronic diseases(counts)	1.76 ± 1.49	1.55 ± 1.42	1.92 ± 1.53	−6.162	0.000
Chronic diseases groups(counts)					
0	555(21.50)	283(26.06)	272(18.19)	42.688	0.000
1–2	1325(51.34)	572(52.67)	753(50.37)		
3–14	701(27.16)	231(21.27)	470(31.44)		
BMI (kg/m^2^)	23.70 ± 4.09	23.86 ± 4.08	23.58 ± 4.09	1.711	0.087
BMI categories					
<18.5	200(7.75)	68(6.26)	132(8.83)	6.831	0.077
18.5–24	1253(48.55)	530(48.80)	723(48.36)		
24–28	791(30.65)	349(32.14)	442(29.57)		
≥28	337(13.06)	139(12.80)	198(13.24)		
Frailty					
No	2281(88.37)	1046(96.32)	1235(82.61)	115.07	0.000
Yes	300(11.62)	40(3.68)	260(17.39)		

**Table 3:** Baseline characteristics classified according to subsequent onset of frail

Variables	2011→2013	*P1*	2011→2015	*P2*
	Incidence rate (N = 1068,%)		Incidence rate (N = 607,%)	
Age(years)		0.016		0.013
45–54	1.59		1.65	
55–64	2.81		2.64	
65–74	3.00		3.46	
≥75	0.94		1.15	
Sex		0.878		0.092
Male	3.00		1.98	
Female	5.34		6.92	
Education		0.001		0.018
Illiterate	4.03		4.61	
Less than elementary school	4.31		3.79	
High school	0.00		0.16	
Above vocational school	0.00		0.33	
Marital status		0.346		0.335
Single	1.40		1.48	
Married	6.93		7.41	
Current residence		0.021	0.00	0.124
Rural	8.15		8.73	
Urban	0.19		0.16	
Current smoking		0.802		0.470
No	5.62		6.92	
Former smoke	0.56		0.49	
Current smoke	2.15		1.48	
Alcohol drinking		0.113		0.122
No	6.93		7.58	
Less than once a month	0.47		0.16	
More than once a month	0.94		1.15	
Taking activities		0.293		0.049
No	4.59		5.77	
Yes	3.75		3.13	
Chronic diseases(counts)		0.008		0.022
0	1.12		0.99	
1–2	3.65		3.79	
3–14	3.56		4.12	
BMI (kg/m^2^)		0.090		0.128
<18.5	1.22		1.32	
18.5–24	3.46		4.12	
24–28	2.72		2.14	
≥28	0.94		1.32	

**Table 4:** Odds ratios (ORs) and 95% confidence interval (CIs) for frailty and components of frailty at baseline associated with Depressive Symptoms at baseline

N = 2581	Model 1 OR (95%CI)	Wald, df	*P*	Model 2 OR (95%CI)	Wald, df	*P*	Model 3 OR (95%CI)	Wald, df	*P*	Model 4 OR (95%CI)	Wald, df	*P*
**Frailty status**												
**Depression**												
No (1086)	Ref (1.000)			Ref (1.000)			Ref (1.000)			Ref (1.000)		
Yes (1495)	5.505(3.907,7.757)	95.042,1	0.000	5.681(3.999,8.087)	94.070,1	0.000	5.284(3.709,7.527)	85.044,1	0.000	5.222(3.665,7.442)	83.691	0.000
**Weakness**												
**Depression**												
No (1086)	Ref (1.000)			Ref (1.000)			Ref (1.000)			Ref (1.000)		
Yes (1495)	2.395(1.803,3.183)	36.271,1	0.000	2.270(1.691,3.049)	29.698,1	0.000	2.072(1.537,2.793)	22.849,1	0.000	2.037(1.510,2.748)	21.708,1	0.000
**Slowness**												
**Depression**												
No (1086)	Ref (1.000)			Ref (1.000)			Ref (1.000)			Ref (1.000)		
Yes (1495)	1.969(1.638,2.368)	51.954,1	0.000	2.270(1.691,3.049)	29.698,1	0.000	1.963(1.653,2.332)	59.061,1	0.000	1.858(1.528,2.259)	38.513,1	0.000
**Weight loss**												
**Depression**												
No (1086)	Ref (1.000)			Ref (1.000)			Ref (1.000)			Ref (1.000)		
Yes (1495)	1.688(1.301,2.189)	15.571,1	0.000	1.668(1.281,2.172)	14.414,1	0.000	1.576(1.206,1.206)	11.113,1	0.001	1.531(1.170,2.004)	9.659,1	0.002
**Exhaustion**												
**Depression**												
No (1086)	Ref (1.000)			Ref (1.000)			Ref (1.000)			Ref (1.000)		
Yes (1495)	12.210(10.035,14.585)	624.471,1	0.000	12.496(10.215,15.286)	603.195,1	0.000	12.210(9.962,14.965)	581.045,1	0.000	12.140(9.903,14.882)	577.203,1	0.000
**Low activity**												
**Depression**												
No (1086)	Ref (1.000)			Ref (1.000)			Ref (1.000)			Ref (1.000)		
Yes (1495)	1.169(0.938,1.457)	1.936,1	0.164	1.218(0.970,1.528)	2.889,1	0.089	1.188(0.943,1.1497)	2.143,1	0.143	1.207(0.957,1.521)	2.531,1	0.112

**Table 5:** Association between Depressive Symptoms and incident frailty not frailty at baseline

Follow-up period		Model 1 HR (95%CI)	Wald, df	*P*	Model 2 HR (95%CI)	Wald, df	*P*	Model 3 HR (95%CI)	Wald, df	*P*	Model 4 HR (95%CI)	Wald, df	*P*
**2011 → 2013**	**Depression**												
**N = 1068**	No (457)	Ref (1.000)			Ref (1.000)			Ref (1.000)			Ref (1.000)		
	Yes (611)	2.148(1.323,3.488)	9.550,1	0.002	2.278(1.378,3.743)	10.562,1	0.001	2.200(1.329,3.641)	9.403,1	0.002	2.193(1.324,3.631)	9.313,1	0.002
**2011 → 2015**	**Depression**												
**N = 607**	No (271)	Ref (1.000)			Ref (1.000)			Ref (1.000)			Ref (1.000)		
	Yes (336)	2.032(1.107,3.731)	5.234,1	0.022	2.096(1.127,3.897)	5.469,1	0.019	1.952(1.036,3.676)	4.286,1	0.038	1.926(1.021,3.632)	4.101,1	0.043

**Table 6:** Odds ratios (ORs) and 95% confidence interval (CIs) frailty and components of frailty at baseline associated with Depressive Symptoms at 2011→ 2013

N = 1068	Model 1 OR (95%CI)	Wald, df	*P*	Model 2 OR (95%CI)	Wald, df	*P*	Model 3 OR (95%CI)	Wald, df	*P*	Model 4 OR (95%CI)	Wald, df	*P*
**Frailty status**												
**Depression**												
No (457)	Ref (1.000)			Ref (1.000)			Ref (1.000)			Ref (1.000)		
Yes (611)	2.148(1.323,3.488)	9.550,1	0.002	2.278(1.378,3.743)	10.562,1	0.001	2.200(1.329,3.641)	9.403,1	0.002	2.193(1.324,3.631)	9.313,1	0.002
**Weakness**												
**Depression**												
No (457)	Ref (1.000)			Ref (1.000)			Ref (1.000)			Ref (1.000)		
Yes (611)	2.003(1.279,3.316)	9.207,1	0.002	2.099(1.325,3.326)	9.967,1	0.002	2.000(1.257,3.181)	8.566,1	0.003	1.990(1.250,3.166)	8.427,1	0.004
**Slowness**												
**Depression**												
No (457)	Ref (1.000)			Ref (1.000)			Ref (1.000)			Ref (1.000)		
Yes (611)	1.375(1.045,1.809)	5.167,1	0.023	1.441(1.084,1.914)	6.343,1	0.012	1.371(1.028,1.829)	4.608,1	0.032	1.395(1.044,1.865)	5.072,1	0.024
**Weight loss**												
**Depression**												
No (457)	Ref (1.000)			Ref (1.000)			Ref (1.000)			Ref (1.000)		
Yes (611)	1.517(1.031,2.232)	4.472,1	0.034	1.430(0.965,2.119)	3.170,1	0.075	1.372(0.922,2.042)	2.429,1	0.119	1.374(0.923,2.046)	2.449,1	0.118
**Exhaustion**												
**Depression**												
No (457)	Ref (1.000)			Ref (1.000)			Ref (1.000)			Ref (1.000)		
Yes (611)	2.878(2.212,3.744)	61.943,1	0.000	2.871(2.194,3757)	59.025,1	0.000	2.842(2.162,3.735)	56.043,1	0.000	2.827(2.150,3.719)	55.223,1	0.000
**Low activity**												
**Depression**												
No (457)	Ref (1.000)			Ref (1.000)			Ref (1.000)			Ref (1.000)		
Yes (611)	0.648(0.455,0.924)	5.755,1	0.016	0.694(0.482,0.998)	3.880,1	0.049	0.684(0.474,0.988)	4.108,1	0.043	0.695(0.481,1.004)	3.758,1	0.053

**Table 7:** Odds ratios (ORs) and 95% confidence interval (CIs) frailty and components of frailty at baseline associated with Depressive Symptoms at 2011→ 2015

N = 670	Model 1 OR (95%CI)	Wald, df	*P*	Model 2 OR (95%CI)	Wald, df	*P*	Model 3 OR (95%CI)	Wald, df	*P*	Model 4 OR (95%CI)	Wald, df	*P*
**Frailty status**												
**Depression**												
No (271)	Ref (1.000)			Ref (1.000)			Ref (1.000)			Ref (1.000)		
Yes (336)	2.032(1.107,3.731)	5.234,1	0.022	2.096(1.127,3.897)	5.469,1	0.019	1.952(1.036,3.676)	4.286,1	0.038	1.926(1.021,3.632)	4.101,1	0.043
**Weakness**												
**Depression**												
No (271)	Ref (1.000)			Ref (1.000)			Ref (1.000)			Ref (1.000)		
Yes (336)	1.107(0.727,1.685)	0.224,1	0.636	1.078(0.701,1.658)	0.116,1	0.733	0.993(0.637,1.548)	0.001,1	0.975	0.983(0.630,1.534)	0.006,1	0.940
**Slowness**												
**Depression**												
No (271)	Ref (1.000)			Ref (1.000)			Ref (1.000)			Ref (1.000)		
Yes (336)	0.916(0.637,1.316)	0.226,1	0.634	0.885(0.610,1.283)	0.418,1	0.518	0.838(0.572,1.229)	0.816,1	0.366	0.845(0.576,1.239)	0.747,1	0.388
**Weight loss**												
**Depression**												
No (271)	Ref (1.000)			Ref (1.000)			Ref (1.000)			Ref (1.000)		
Yes (336)	1.620(0.866,3.029	2.282,1	0.131	1.767(0.929,3.363)	3.009,1	0.083	1.926(0.998,3.714)	3.821,1	0.051	1.503(0.666,3.393)	0.963,1	0.326
**Exhaustion**												
**Depression**												
No (271)	Ref (1.000)			Ref (1.000)			Ref (1.000)			Ref (1.000)		
Yes (336)	2.904(2.052,4.111)	36.149,1	0.000	2.921(2.056,4.149)	35.819,1	0.000	2.864(2.004,4.094)	33.309,1	0.000	2.869(2.004,4.109)	33.122,1	0.000
**Low activity**												
**Depression**												
No (271)	Ref (1.000)			Ref (1.000)			Ref (1.000)			Ref (1.000)		
Yes (336)	0.823(0.519,1.306)	0.683	0.409	0.856(0.535,1.370)	0.419,1	0.518	0.834(0.518,1.345)	0.552,1	0.458	0.839(0.520,1.353)	0.517,1	0.472

## Data Availability

The data that support the findings of this research are available from the public, open-access website (https://charls.pku.edu.cn/).

## Abbreviations

CHARLS: China Health and Retirement Longitudinal Study
CAPI: computer-aided personal interview
HRs: hazard ratios
OR: odds ratio
BMI: body mass index
kg/m^2^: kilogram/meter^2^
M: mean
SD: standard deviation
CIs: confidence intervals
CESD: Chinese version of the Center for Epidemiologic Studies-Depression Scale
IL-6: interleukin-6
ATP: adenosine triphosphate
NSFC: The National Natural Science Foundation of China
NIA: National Institute on Aging
WB: World Bank
